# Patients’ Perception of Follow-Up Care and Personal Health Status of 677 Long-Term Survivors of Gynecological Cancer from the Study “Expression IX—Long-Term Survival with Gynecological Cancer”: The International NOGGO, ENGOT and GCIG Survey

**DOI:** 10.3390/cancers18101647

**Published:** 2026-05-20

**Authors:** Hannah Woopen, Tibor Zwimpfer, Luise Brenner, Clemens Liebrich, Katharina Leitner, Stephanie Henry, Cornelia Müller, Flurina Annacarina Maria Saner, Christoph Ebner, Desislava Dimitrova, Claudia Mang, Isabelle Himsl, Johanna Hell-Teutsch, Toon Van Gorp, Christian Braun, Yurtcu Nurhayat, Michael Müller, Lars Hanker, Viola Heinzelmann-Schwarz, Jalid Sehouli

**Affiliations:** 1 Department of Gynecology with Center for Oncological Surgery, Charité—Universitätsmedizin Berlin, Corporate Member of Freie Universität Berlin and Humboldt-Universität zu Berlin, 13353 Berlin, Germany; 2 North-Eastern German Society for Gynecological Oncology (NOGGO), 13359 Berlin, Germany; 3 Swiss GO Trial Group (Swiss-GO), 4031 Basel, Switzerland; 4 Gynecological Cancer Centre, University Hospital Basel, 4031 Basel, Switzerland; 5 Ovarian Cancer Research, Department of Biomedicine, University of Basel, 4031 Basel, Switzerland; 6 Gynecological Cancer Center, Wolfsburg Hospital, Sauerbruchstrasse 7, 38440 Wolfsburg, Germany; 7 Department of Gynecology and Obstetrics, Medical University of Innsbruck, 6020 Innsbruck, Austria; 8 Arbeitsgemeinschaft Gynaekologische Onkologie Austria (AGO Austria), 6020 Innsbruck, Austria; 9 Belgium and Luxembourg Gynaecological Oncology Group (BGOG), 3000 Leuven, Belgium; 10 Oncology, CHU UCL NAMUR (Site Ste Elisabeth), Université Catholique de Louvain, 5000 Namur, Belgium; 11 Department of Gynecology and Obstetrics, Brandenburg University Hospital GmbH, 14770 Brandenburg an der Havel, Germany; 12 Department of Gynaecology and Obstetrics, Bern University Hospital, Faculty of Medicine, University of Bern, 3010 Bern, Switzerland; 13 Department of Gynecology and Obstetrics, Third Order Hospital Munich, 80638 Munich, Germany; 14 Department of Gynecology and Obstetrics, Wels-Grieskirchen Hospital, Grieskirchner Straße 42, 4600 Wels, Austria; 15 Leuven Cancer Institute, Catholic University of Leuven, 3000 Leuven, Belgium; 16 Luzerner Kantonsspital Spitalstrasse, 6000 Luzern, Switzerland; 17 AGAPLESION Klinikum Hagen gGmbH, Academic Teaching Hospital of the Ruhr University Bochum, Grünstraße 35, 58059 Hagen, Germany; 18 Department of Gynecology and Obstetrics, University Hospital Münster, Albert-Schweitzer-Campus 1, 48149 Münster, Germany

**Keywords:** cancer survivorship, long-term survivors, gynecological cancer, long-term side effects, secondary cancer

## Abstract

**Background:** Long-term survivors (LTS) after gynecological cancer may be cured but still face physical and psychological challenges. This multicenter study aimed to assess the long-term side effects, the received follow-up care, and the personal perspectives of survivors. **Methods:** Between 2019 and 2025, LTS from four European countries within the ENGOT (European Network of Gynecological Oncological Trial Groups) and GCIG (Gynecologic Cancer InterGroup) networks were recruited. Long-term survival was defined as surviving at least five years after the first diagnosis. LTS completed a questionnaire with 81 questions (patient’s characteristics, oncological history, current health status, lifestyle factors). Analyses were mainly descriptive. **Results:** A total of 677 LTS were enrolled, with a median age of 64.0 years (range: 26–92) and a median survival time of 7 years (range: 5–38). A total of 46.6% were diagnosed with cervical cancer, 32.9% with endometrial cancer, 4.4% with ovarian cancer, and 16.1% with other types of gynecological cancer. Moreover, 36.9% still suffer from physical and psychological symptoms, most frequently being lymphedema (36.2%), hot flashes (22.4%), difficulties with concentration (21.1%), fatigue (20.9%), vaginal dryness (20.1%), and urinary incontinence (18.9%). Median overall health status was ranked (scale 1–5; 1 = very good, 5 = very poor) as 2, while 13.5% rated their health as poor/very poor. Current symptoms were associated with poorer health status (*p* < 0.001) and a history of recurrent disease (*p* = 0.001). In addition, 13.6% reported not receiving follow-up care. CA-125 was determined in 80.8% of ovarian LTS, as well as in 30.7% of cervical and 28.9% of endometrial LTS. Pap smear follow-up was reported by 50.5% of endometrial LTS. A total of 33.7% did not exercise at all or exercised less than an hour per week, 13.4% smoke tobacco, and 51.2% drink alcohol more often than once a month. **Conclusions:** Our findings highlight the need for patient-centered follow-up care, addressing both long-term side effects and education on lifestyle and prevention. Follow-up procedures that do not follow guidelines should be avoided.

## Simple Summary

This study aimed to improve understanding of gynecological cancer survivors, with a focus on patients’ perspectives on persistent long-term therapy- and tumor-related symptoms, as well as the evaluation of follow-up procedures and the characterization of patients’ and their overall lifestyles. Long-term survivors, defined as individuals who have survived at least five years since their initial diagnosis, completed an 81-question survey. Overall, 677 patients were enrolled, with a median survival time of 7 years. They reported various ongoing symptoms, including lymphedema (36.2%), hot flashes (22.4%), difficulties with concentration (21.1%), fatigue (20.9%), vaginal dryness (20.1%), and urinary incontinence (18.9%). Additionally, Follow-up procedures that do not follow the guidelines were stated. These results underscore the need for patient-centered follow-up care to address ongoing symptoms and provide lifestyle advice, which could help improve overall health and establish standard, long-term follow-up care to avoid unnecessary procedures.

## 1. Introduction

Worldwide, every year, 661,021 women are newly diagnosed with cervical cancer, 420,242 with endometrial cancer, and 324,398 with ovarian cancer [1]. Due to improved screening programs for cervical cancer and improved patient-tailored treatment, including new evolving therapies, the number of long-term survivors (LTS) after gynecological cancer is continuously growing [2]. Overall, according to data from the SEER database, approximately 81.1% of patients with uterine cancer, 68.0% with cervical cancer, and 51.6% with ovarian cancer survive for at least 5 years and become LTS according to the definition of the GCIG consensus guideline, where long-term survival after gynecological cancer was defined as survival of at least five years from first cancer diagnosis [3,4,5,6].

Despite the rising number of survivors, few studies attempt to systematically identify psychological and physical problems that LTS may still suffer from. Westin et al. reported that fatigue, sleep disturbance, urinary difficulties, sexual dysfunction, neurological issues, bowel complaints, depression, and memory problems are the most common health issues among gynecological cancer survivors. However, the transferability of the data to LTS is limited due to the fact that the study group included survivors from within months after first diagnosis (median survival time: 4.9 years (range: 0.1–57.6)) [7]. Nevertheless, these findings show the importance of further research regarding persistent long-term symptoms. Similarly, the international study Expression VI–Carolin Meets HANNA, which included 1044 ovarian, tubal, and peritoneal long-term cancer survivors, showed that almost half continued to experience persistent treatment-related side effects [8]. Most frequently reported symptoms were lymphedema (37.7%), fatigue (23.9%), pain (21.6%), polyneuropathy (16.9%), gastrointestinal problems (16.6%), and memory problems (15.5%). These findings raise the question of whether LTS with other gynecological tumors are affected by similar challenges.

In addition to diagnosing and managing the symptoms of long-term side effects, survivorship care should also include primary and secondary prevention, as well as lifestyle counseling, in order to improve quality of life, cardiovascular health, and reduce the risk of secondary cancer [9,10]. Especially of the latter, there is good evidence that the risk for cardiovascular disease and secondary cancer increases with survival years [11]. This is why follow-up and survivorship should not only cover the first five years after diagnosis [3].

Lifestyle counseling and patient empowerment are a crucial part of survivorship care. Lifestyle factors are associated not only with quality of life but also with long-term outcomes. Bian et al. already showed an influence on all-cause mortality of all types of cancer survivors by avoiding smoking, light alcohol consumption, adequate physical activity, a healthy diet, and optimal BMI [12]. Especially, endometrial cancer patients frequently have many cardiovascular risks, so survivorship care should include cardiovascular disease prevention and management, as stated in the updated ESGO guideline for endometrial cancer [13]. Lifestyle counseling is not only important for endometrial cancer survivors but for all gynecological cancer survivors. In cervical cancer survivors, it could be shown that lifestyle counseling, including nicotine cessation programs, could improve quality of life and reduce distress, anxiety, and depression rates [14]. Smoking is a known risk factor for secondary cancer after cervical cancer [15]. Regarding secondary cancer and physical health, patients shall be encouraged to participate in national screening programs and follow national guidelines according to their tumor entity, such as mammograms every two years after ovarian cancer [3,16,17].

As shown, LTS face a broad range of challenges. To address this, survivorship care has recently been implemented into the new ESGO guidelines, which also provide a minimum checklist for a structured approach. This includes the previously mentioned points, such as screening, prevention, and treatment of persistent symptoms, as well as psychosocial aspects such as distress. It also includes the prevention of new secondary malignancies and secondary and tertiary prevention, particularly with regard to cardiovascular diseases, and surveillance for recurrence. But still, in everyday practice, follow-up care is very heterogeneous, and many LTS do not have access to structured and multiprofessional survivorship care [3]. This is partly because most cancer trials focus on follow-up within the first years after diagnosis, and long-term data are lacking. Only small cohorts of LTS with gynecological cancer have been published regarding patients’ characteristics, which is important to understand this growing and interesting patient group, for example, to identify prognostic factors for long-term survival. Especially for LTS living more than five years after cancer diagnosis, there are few studies on patients’ perspectives on long-term side effects, lifestyle factors, and their needs.

Taken together, the study aimed to (1) characterize LTS with gynecological cancer, (2) identify persistent tumor- and therapy-related symptoms, (3) evaluate received follow-up care, and (4) describe reported lifestyle factors.

## 2. Materials and Methods

The international survey “Longterm survival with gynecological cancer—Expression IX” was initiated by the North-Eastern German Society of Gynecological Oncology (NOGGO) and the Department of Gynecology in the Center for Oncological Surgery of the Charité—Universitätsmedizin Berlin. As part of the multicenter study, long-term survivors (LTS) of all types of gynecological cancer were included, irrespective of type, current stage, or therapy. Recruitment took place between 2019 and 2025 in four different countries, available for members of ENGOT (European Network of Gynecological Oncological Trial Groups) and GCIG (Gynecologic Cancer InterGroup): Austria (AGO Austria—Arbeitsgemeinschaft Gynaekologische Onkologie Austria), Belgium (BGOG—Belgium and Luxembourg Gynecological Oncology Group), Germany (NOGGO—North-Eastern German Society of Gynecological Oncology), and Switzerland (Swiss GO Trial Group). The number of centers participating was as follows: 7 in Austria, 8 in Belgium, 30 in Germany, and 12 in Switzerland.

Based on the data collection from Expression VI—Carolin meets HANNA, which included long-term ovarian cancer survivors, Expression IX aimed to expand the cohort of LTS. Additionally, patients with cervical cancer, endometrial cancer, and other rare gynecological tumors (e.g., gynecological sarcomas of the uterus, vaginal or vulva carcinoma) were included. According to GCIG consensus guidelines, long-term survival is defined as survival of at least five years from the first diagnosis [3].

The survey was translated into four different languages (German, English, French, and Italian) by certified translators and additionally validated by bilingual physicians to ensure its accuracy. A pilot phase was conducted to test the survey for readability and understandability in a small patient cohort (n = 10). As the Distress Thermometer had been validated and most of the questions had already been tested on a large patient cohort in previous Expression surveys, the readability and understandability were confirmed by all patients in the pilot phase. Ethical approval was obtained from ‘Charité—Universitätsmedizin Berlin’ in Germany (AVD-No: EA2/181/18).

The inclusion criteria were defined as follows:

- Patients were >18 years.
- Gynecological cancer diagnosis was at least 5 years ago.
- A willingness to complete the questionnaire.

There were no exclusion criteria. An active cancer treatment, or recurrent cancer, did not exclude participants.

Recruitment took place during follow-up visits at gynecological oncology departments and general practitioners. Furthermore, the study was distributed to patient conferences, self-help groups, and patient journals. Overall, LTS were identified in hospitals and in the ambulatory sector.

To answer the survey, patients could use the online version or the paper-based questionnaire. The survey was anonymous. No signed consent was required; the patient’s consent is assumed when completing the questionnaire. In addition, patients were informed in advance about the objectives of the survey in an accompanying letter.

There were 81 questions, mainly closed single-choice questions, followed by multiple-choice questions and ranking or rating questions. Overall, there were only a few open questions included. The questionnaire can be divided into three parts: The first part included questions about patients’ characteristics (e.g., age, height, weight, marital status, children) and specific tumor characteristics (e.g., cancer entity, stage, treatment, recurrences). The second part dealt with current health status (e.g., specific side effects during and after treatment and their ongoing impact on life, other known diseases, ranking of distress, and overall condition, received follow-up care). The presence of current symptoms was recorded using a binary yes/no question. The ranking of distress was measured using the validated Distress Thermometer, a visual analog scale ranging from 0 to 10, where higher scores indicate greater distress [18]. The overall health condition was measured using a Likert scale from 1 to 5 (1 = very good, 5 = very poor).

The third part described health competence, for example, regarding self-perception as a cancer patient, physical activity, alcohol consumption, tobacco smoking, self-reported sexual activity, and interest in sex. Most questions were those used in previous NOGGO Expression studies, with a few adjustments made to adapt the expanded study cohort. The whole questionnaire is available on the survey’s website (https://redcap.charite.de/cru/surveys/?s=AJKEMYD4L3P9DLMW) (accessed on 17 February 2026). Apart from the questions from previous Expression studies, which involved more than 14,000 participants, and the NCCN Distress Thermometer, no other validated quality-of-life questionnaire was used.

Variations in n can be explained due to missing data, as not all participants completed the survey in full. The percentages in Section 3 refer to the number of respondents for each item; n always indicates the total number of participants for the given question.

The statistical analyses were performed using SPSS software (Statistical Package for the Social Sciences), Version: 31.0.0.0 (117). Descriptive statistics were used, showing frequencies and percentages for categorical data and describing continuous data using means, median, and ranges. A Chi-squared test was used to show statistical comparisons. Linear and logistic regression analyses were calculated for outcomes such as health status, distress, and long-term side effects. The analysis was exploratory, and the *p*-values below 0.05 were considered as signals of potential effects.

## 3. Results

### 3.1. Patient Characteristics

From November 2019 to September 2025, a total of 677 long-term survivors (LTS) with gynecological cancer have participated in the Expression IX study. Overall, patients were diagnosed with the following gynecological cancers: 277 women with cervical cancer (46.6%), 196 (32.9%) with endometrial cancer, 26 (4.4%) with ovarian, tubal, or peritoneal cancer, and 96 (16.1%) with other types of cancer. The ‘others’ category included 13 patients with granulosa cell tumors, 18 patients with vulvar cancers, five patients with vaginal cancers, 31 patients with gynecological sarcomas, and six patients with breast cancer, among others not specified (23 patients).

The median age was 64.0 years (range: 26–92 years, SD = standard deviation 12.5) at study entry, while the median age at diagnosis was 55.0 years (range: 14 to 86 years, SD 13.0). The median survival time at recruitment was 7 years (range: 5–38 years). Of the surveyed women, 75.8% had survived for 5 to 10 years, while 24.2% had survived more than 10 years (n = 375). The patients’ characteristics are shown in Table 1.

Among patients with known FIGO stage, most were diagnosed with FIGO I/II at initial diagnosis (31.9%), while 10.3% were diagnosed with FIGO stage III/IV. However, more than half of the patients (57.9%) did not know their FIGO stage at diagnosis (n = 508). Among those with a known FIGO stage, significantly more ovarian cancer patients were diagnosed with advanced FIGO stages III/IV (78.6%) compared with cervical (22.8%), endometrial (14.7%), or other cancer patients (16.7%), *p* < 0.001.

About two-thirds were married or lived in a cohabiting relationship (59.2%). Moreover, 71.7% of the women had children, most at least two (70.6%). The mean number of children was 2.01, ranging from one to six children. Most patients received support in coping with cancer (91.8%), mainly provided by family members (53.3%) and doctors (31.9%). Most participants had completed a lower secondary education (33.1%), followed by a university degree (20.9%), intermediate secondary education (19.8%), education categorized as ‘others’ (13.3%), upper secondary education (9.7%), and no formal education (3.2%). Overall, 56.0% of the women were retired, 37.9% were (self-)employed, and 6.1% were unable to work or unemployed.

At the time of initial diagnosis, 76.5% had no metastases, 10.0% had metastases, and 13.5% could not remember (n = 600). The majority underwent surgery after the first diagnosis (93.9%). About two-thirds received chemotherapy (61.9%), radiotherapy was performed in 39.4%, and 9.1% underwent maintenance therapy. Although 21.2% developed recurrent disease (n = 130/613), most of them had recurrence ≥12 months after their last chemotherapy (69.6%, n = 78/112). In the event of recurrence, another surgery was performed in 68.8% of the cases (n = 86/125). A total of 34.9% of the patients with a history of recurrent disease experienced more than one relapse.

At the time of the survey, the majority of women were free of recurrence (88.2%), while 4.1% had active recurrence and 7.7% were receiving cancer treatment (n = 558).

### 3.2. Health Status and Long-Term Side Effects

**Figure 1:**
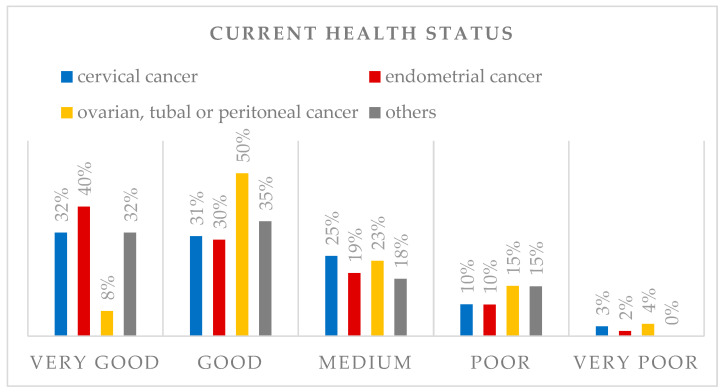
Health status rated on a scale of 1–5 (1 = very good to 5 = very poor) and differentiation between the different tumor entities.

Overall health status (Figure 1) was rated very good or good by two-thirds of the participants (64.0%). Whereas 13.5% described it as poor or very poor. The median health status on the scale from 1 (very good) to 5 (very poor) was 2 (n = 608).

Recurrence-free patients rated their health status as very good (37.4%) or good (31.1%) most frequently, compared to patients who had a recurrence, who mainly rated their health status as medium (37.5%) or good (35.9%), *p* < 0.001. The median health status for patients without a history of recurrence is 2, compared to 2.5 for patients with a history of recurrence.

In total, 63.1% of the patients did not report any current symptoms, while 36.9% of patients still had symptoms. Overall, 37.7% of cervical cancer patients, 33.3% of endometrial cancer patients, 32.1% of other cancer patients, and 62.5% of ovarian, tubal, and peritoneal cancer patients reported current symptoms. There was a significant association between a tumor entity and the presence of current symptoms (*p* = 0.035). Current symptoms were significantly more frequently reported from patients still receiving oncological treatment (53.5%) and active recurrence (68.2%) compared to recurrence-free cancer patients (34.7%). The presence of current symptoms was associated with poorer overall health status (*p* < 0.001). There was also an association of current symptoms with a history of recurrent disease (*p* = 0.001). In linear regression analysis, there was no association of health status and presence of current symptoms found with age, survival years, tumor entities, recurrent status, current disease status, and therapy modalities. Women interested in sex and being sexually active were less likely to complain of side effects than those not interested in sex (*p* = 0.003). No association was found between symptoms and the overall time of survival or between different age groups.

### 3.3. Symptoms and Diseases

**Figure 2:**
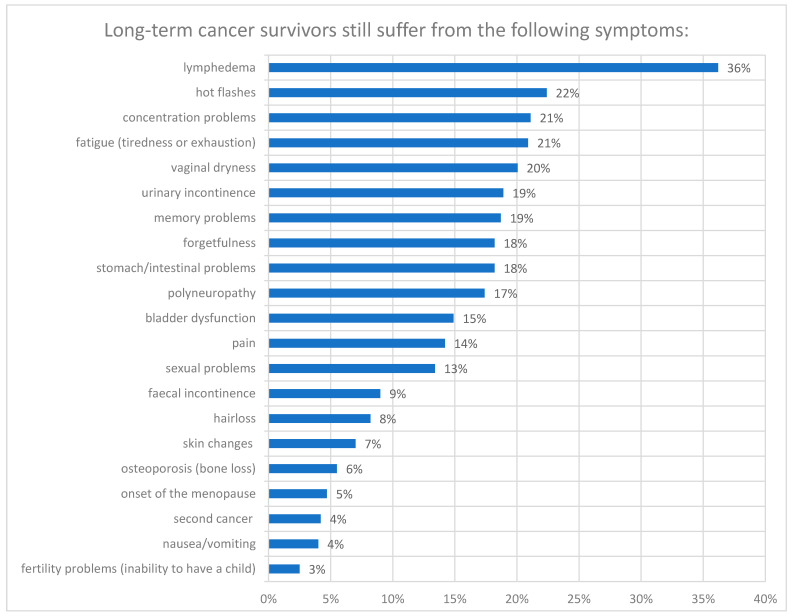
Symptoms that LTS with gynecological cancer still suffer from.

The following side effects occurred most frequently during treatment: severe fatigue (40.7%), hair loss (40.0%), vaginal dryness (33.7%), peripheral neuropathy (32.8%), and hot flashes (32.0%). Patients reported that they were still suffering from the following symptoms after a mean time of 9.24 years after initial diagnosis: lymphedema (36.2%), hot flashes (22.4%), concentration problems (21.1%), fatigue (20.9%), vaginal dryness (20.06%), and urinary incontinence (18.9%). Figure 2 shows all the long-term side effects that were reported by the patients.

With a closer look at different tumor entities, women with cervical cancer stated they are still struggling most with lymphedema (39.4%), urinary incontinence (25.6%), bladder dysfunction (23.3%), memory problems, stomach/intestinal problems, and hot flashes (22.7% each). While patients with endometrial cancer reported most frequently lymphedema (30.1%), vaginal dryness (26.4%), other unspecified symptoms (21.7%), and hot flashes (20.8%). Almost 50% of patients with ovarian/tubal/peritoneal cancer most frequently reported lymphedema (46.2%), followed by hot flashes (40.0%), peripheral neuropathy and stomach/intestinal problems (36.0% each), and memory problems and forgetfulness (32.0% each). Patients with tumors assigned to the category ‘others’ most commonly reported lymphedema (35.1%), fatigue (27.3%), hot flashes (22.7%), and peripheral neuropathy (19.7%).

Symptoms that were reported by more than 20% of recurrence-free cancer survivors were: lymphedema (34.6%), vaginal dryness (21.6%), urinary incontinence (21.6%), and hot flashes (20.8%). Among patients with a history of recurrence, the following eight symptoms were reported by more than 20% of patients: lymphedema (43.7%), fatigue (33.7%), polyneuropathy (30.6%), concentration problems (29.6%), memory problems (28.6%), hot flashes (26.5%), forgetfulness (26.5%), and stomach/intestinal problems (25.5%). Overall, patients with a history of recurrence reported more symptoms.

In addition, 8.7% of patients reported a second malignancy, and 4.2% of the reported still suffer from it today. Although 41 individuals confirmed a second malignancy, a total of 45 responses regarding the cancer type were recorded, indicating that some participants reported more than one secondary cancer. The most frequently reported secondary cancer was breast cancer (48.6%), followed by cancers categorized as ‘others’ (35.1%), and skin and colorectal cancers (each 18.9%).

Exploratory multivariable logistic regressions were performed to identify factors that were associated with reported symptoms. Survival years were not associated with distress level, health status, or specific long-term side effects. Clinical factors that were associated with specific long-term side effects were age and recurrence status.

The most prevalent comorbidity before initial diagnosis was hypertension (47.0%). Notably, the depression rate increased from 11.9% to 20.8% after cancer diagnosis.

### 3.4. Psychological Aspects

Distress was assessed using the Distress Thermometer on a scale from 0 to 10. In clinical routine, a psycho-oncological consultation is recommended with a score ≥6 on the Distress Thermometer. The mean distress score was rated as 3.7 (median 3, SD 3.0). Patients with cervical cancer reported a mean distress score of 4.1 (SD 3.1), compared to 3.2 (SD 2.8) for patients with endometrial cancer, 4.6 (SD 2.5) for patients with ovarian, tubal, and peritoneal cancer, and 3.4 (SD 2.8) for patients with other types of cancer (*p* = 0.003). Post hoc analyses showed that cervical cancer patients had significantly higher distress scores than endometrial cancer patients. In addition, ovarian, tubal, and peritoneal cancer patients had higher distress scores than endometrial cancer patients. No significant differences were found between the other groups and their mean distress scores. In addition, the mean distress score for recurrence-free patients was 3.5, compared to 4.5 for patients who had experienced a recurrence. In linear regression analyses, distress was associated with younger age (OR 0.059, 95% CI 0.083–0.036, *p* < 0.001).

**Figure 3:**
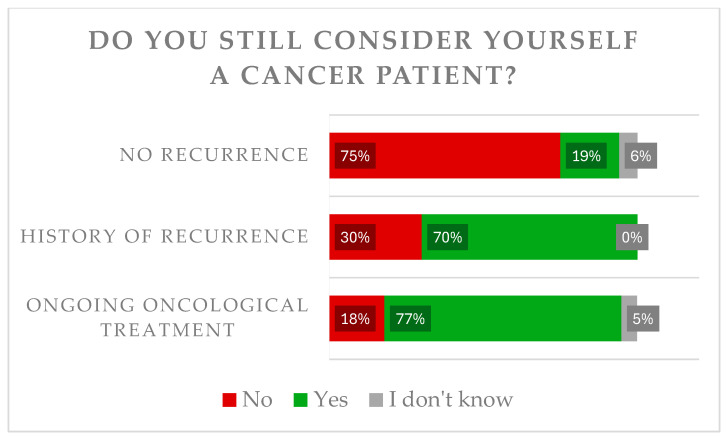
Depiction whether patients regard themselves as a cancer patient according to their disease status.

A quarter still regarded themselves as cancer patients (25.5%), while 68.3% disagreed with this thesis, 6.2% were not sure (n = 597). A total of 76.9% who are still receiving therapy and 70.0% who are currently having a recurrence regard themselves as cancer patients, compared to 19.1% who are living recurrence free (*p* < 0.001) (Figure 3). Overall, 59.5% of those who had experienced a recurrence of the disease still considered themselves to be cancer patients.

Patients who are still suffering symptoms regard themselves more often as cancer patients than those without symptoms (41.4% vs. 17.6%), *p* < 0.001. There was no association between the survival time and the identification as cancer patients (5–10 years: 23.6% vs. >10 years 28.8%).

### 3.5. Follow-Up Care

Table 2 depicts follow-up procedures. A total of 13.6% of the participants reported not receiving any follow-up care at all. Receiving a medical consultation was reported by 43.8%, and almost half indicated undergoing a physical examination (49.7%). When stratified by a tumor entity, the largest subgroup without any follow-up care was observed among patients with cervical cancer (16.9%), followed by endometrial (12.1%), ovarian/tubal/peritoneal (7.7%), and “others” (5.7%).

Serum tumor marker CA-125 was determined in 80.8% of the cases with ovarian/tubal/peritoneal cancer, as well as in 49.4% of the cases categorized as ‘other’ cancer subtypes. Interestingly, 30.7% of cervical and 28.9% of endometrial cancer patients reported CA-125 measurement for monitoring.

Overall, vaginal ultrasound was performed in 62.7% (cervical, 62.2%, endometrial 68.4%, ovarian/tubal/peritoneal 50.0%, and others 57.5%) and abdominal ultrasound in 29.4% (cervical 28.5%, endometrial 30.0%, ovarian/tubal/peritoneal 38.5%, and others 25.3%) of the cases as part of follow-up care.

Out of all the patients, 40.6% of the women reported receiving a regular PAP smear. Among these, the highest rate was observed in women with endometrial cancer, with 50.5% (cervical 37.8%, ovarian/tubal/peritoneal 26.9%, and others 41.4%). Interestingly, patients who underwent surgery and removal of the uterus continued to receive a Pap smear in 40.7% of the cases.

Focusing on breast health, clinical breast examination was reported by 52.8%, and a breast ultrasound and/or mammography was reported by 40.4%.

Additional regular imaging (e.g., MRI, CT, PET, chest X-ray, bone scintigraphy) was performed in 24.4% of participants. Compared to all cancer groups, patients with ovarian/tubal/peritoneal cancer were more likely to undergo further imaging (53.8%). Still, 23.6% of cervical and 16.8% of endometrial cancer patients reported getting regular imaging follow-ups. Patients with a history of recurrence received more frequent lab work, including CA-125 (63.6%) and regular imaging (55.6%).

### 3.6. Physical Activity, Smoking, Alcohol, and Sex

In total, 20.6% did not exercise at all, whereas 65.9% exercised at least one hour up to more than 4 h a week. Another 13.6% exercised less than one hour (n = 583). Since their diagnosis, 25.5% indicated an increase in their weekly activity (n = 604), and 55.1% indicated that they believe physical activity has a positive influence on the progression of their disease. Another 28.6% are unsure about this, and 16.3% disagree. Smoking tobacco was reported by 13.4% of respondents. Patients with cervical cancer form the largest group with 19.7%. Almost half of the participants stated to never drink alcohol or only drink once or less than once per month (48.8%). Therefore, 7.4% reported drinking alcohol more than four times per week. A decrease in alcohol consumption since the cancer diagnosis was reported by 12.6%.

Comparing patients with and without recurrence in terms of active lifestyle, current smoking habits, and alcohol consumption, no significant differences were found (*p* = 0.08, *p* = 0.354, *p* = 0.235, respectively).

Interest in sex and sexual activity was expressed by 37.8% of the participants (n = 439). Patients who experienced a recurrence were less interested in sex, or in having sex at all, than those who were recurrence-free: 28.9% compared to 40.5% (*p* = 0.033). Meanwhile, 25.1% indicated interest in sex but were not sexually active (n = 383). Overall, 85.3% reported being satisfied with their relationship. Interest in further investigations or studies on LTS is shown by 57.6% of the participants.

## 4. Discussion

Our international study included 677 long-term survivors (LTS) of gynecological cancers (cervical, endometrial, ovarian cancer, and other gynecological cancers) with a median survival since diagnosis of 7 years. The median rating of overall health status was 2 (on a scale from 1 (very good) to 5 (very poor)). More than one-third of survivors still experienced long-term side effects such as lymphedema, hot flushes, and concentration problems, and 8.7% of women reported secondary cancers. The follow-up care that was provided ranged from none at all (13.6%) to unnecessary treatments that are not supported by current guidelines, such as Pap smears for patients after ovarian and endometrial cancer [19]. This exploratory analysis revealed indications of significant differences. *p*-values should be interpreted on an exploratory basis. It should be kept in mind that all results are patient-reported.

With an overall increasing number of LTS, it is necessary to take a closer look at the complex needs of this patient group, as many professional associations have already requested [3]. Our findings confirm that long-term side effects among gynecological cancer survivors exist even many years after diagnosis. The symptom burden differs between tumor types, with lymphedema being the most frequently reported long-term side effect within the whole cohort, followed by urinary incontinence for cervical cancer, vaginal dryness for endometrial and hot flashes for ovarian cancer patients. Long-term side effects highly affect the quality of life of patients and, therefore, should be addressed in follow-up care [20]. One very severe late effect is the development of secondary cancer.

This is particularly relevant as 8.7% of our cohort reported a secondary malignancy. These findings are consistent with previous reports indicating an increased risk of secondary cancers compared to the general population [16]. For example, Arnold et al. reported a 5.6% incidence of secondary cancers in cervical cancer survivors, while Woopen et al. reported incidences of 7.6% in ovarian cancer patients [15,17]. BRCA mutations, which may be found in 10–20% of ovarian cancer patients, with even higher rates of BRCA mutations in the LTS group, increase the risk for breast cancer. Within the ovarian and endometrial cancer group, patients with lynch syndrome (MLH1, MSH2, MSH6, PMS2) can also be found [21,22]. Consequently, patients with ovarian cancer and patients with specific histological types of endometrial cancer are currently offered genetic testing and counseling. Since those tests have only been widely available in the last ten years, it is possible that a genetic predisposition and therefore an increased risk of a secondary cancer are not known in LTS. In our cohort, 24.2% of cancer survivors have survived for more than 10 years. With the knowledge that individual risk profiles exist, genetic testing and counseling can be offered as part of long-term follow-up care in order to recommend extended individual preventive examinations such as mammograms/breast ultrasound and colonoscopies. For the whole group of cancer survivors (not only those with genetic predisposition), cancer-screening programs following the national guidelines (such as mammography, melanoma screening, and colonoscopies) are also recommended. After ovarian cancer, breast diagnostics are recommended every two years, regardless of age [23].

In cervical cancer patients, smoking and radiotherapy increased the risk of secondary cancer compared to the general population [15]. This is why smoking cessation is an important part of follow-up care after cervical cancer. After abdominal radiation, colonoscopy is recommended every five years and skin cancer screening every year [24].

Another study regarding cervical cancer survivors revealed an improvement in quality of life, with positive changes in health behaviors, as well as a higher risk of depression and distress, and a lower quality of life for those with adverse health behaviors [14]. In our cohort, cervical cancer patients made up the largest group of smokers at 19.7%. Addressing these lifestyle factors during follow-up care is necessary [25], particularly since Driessen et al. reported that gynecological cancer survivors are unlikely to change their lifestyle after diagnosis [26]. This is particularly important for endometrial cancer survivors, for whom an increased risk of cardiovascular disease has been identified [27,28]. Lifestyle factors that decrease the risk of heart disease are well known and include a well-balanced diet, physical activity, a healthy weight, no alcohol consumption, and quitting smoking.

Since the publication of the randomized Phase 3 trial study with colon cancer patients after adjuvant chemotherapy showed that a three-year structured exercise program resulted in longer disease-free and overall survival, we should strengthen our education about physical activity even more [29]. This is particularly important given that our findings showed that 20.1% of patients did not exercise at all and 13.6% exercised for less than an hour per week. Furthermore, only around half (55.1%) of survivors believe that physical activity has a positive influence on the progression of their disease, compared to 44.9% who are unsure or disagree.

In conclusion, providing tailored lifestyle advice could help improve the health situation, survival time, and reduce the risk of adverse events [30,31]. It is also important to screen those patients at risk and treat comorbidities accordingly.

Our data highlight gaps in education and disease understanding. More than 90% of participants have not been in contact with a survivorship clinic. As reported by Weaver et al., only 41.7% of cancer survivors received information from their physician regarding long-term side effects and side effects of cancer treatment [32]. Similarly, 57.9% of our patients were unable to name their FIGO stage at diagnosis. Implementing structured patient education, starting at the time of diagnosis, may enhance patients’ understanding of their disease and support a more effective recognition and management of long-term symptoms. Weaver et al. reported that 45.7% of endometrial cancer and 20.5% of ovarian cancer survivors had not attended any follow-up visits in the past two years [32]. Since the study collected data before 2007, the lower proportion compared to patients without follow-up care in our cohort (13.6%) may reflect an improvement over time.

More than 85% in our cohort still received follow-up care following the recommendation of lifelong follow-up care, according to the GCIG consensus guideline [3]. However, the reported follow-up procedures should be questioned, as 30.7% of cervical cancer patients and 28.9% of endometrial cancer patients received a CA-125 serum test, even though there is no evidence for these tumor entities. Guidelines do not recommend these procedures [31,33]. Even for ovarian cancer patients, it is known that regular CA-125 testing does not improve overall survival but is associated with a decreased quality of life [23]. However, CA-125 testing can be an additive marker for suspected recurrence in patients with ovarian cancer and is therefore often implemented in clinical routine. In our patient cohort, 50.5% of endometrial cancer survivors and 26.9% of ovarian cancer survivors receive regular Pap smears, which is without any clinical benefit or scientific evidence [31,34]. The latest recommendations are not to perform Pap smears during follow-up in ovarian and endometrial cancer after a hysterectomy [19]. These findings raise questions about whether these examinations create a false sense of security and unnecessarily invade the privacy of our patients, not to mention concerns about unnecessary healthcare costs. The recently published TOTEM study supports the thesis of reconsidering follow-up care, particularly with regard to these issues. The study revealed that an intensive follow-up regimen, including regular vaginal cytology, laboratory tests, or imaging investigations, does not improve the five-year overall survival rate in patients with endometrial cancer when compared to a minimal follow-up regimen [35].

### 4.1. Limitations

This large, multicenter, international study observed a cohort of 677 LTS of gynecological cancers. Due to the variety of cancer types included, there was a wide range of possible treatments, which may result in different long-term side effects. In addition, the size of the cancer subtype cohorts varied and was, in some cases, small. This reflects the varying incidence and survival rates of gynecological cancers. There was a different distribution of FIGO stages among the tumor entities: patients with ovarian cancer were more frequently diagnosed with higher FIGO stages, which is consistent with later detection, compared to tumor entities with screening programs. However, as more than half of the participants could not recall their FIGO stage, analyses regarding FIGO stage were limited. Furthermore, the ‘others’ category combined a heterogeneous group of rare cancer subtypes, which were analyzed together despite their differences. Recruitment is ongoing, and members of the ENGOT and GCIG networks will include more LTS with gynecological cancer in future research, especially those with rare types of cancer. Many LTS do not receive routine follow-up care, which is why it is difficult to recruit a representative sample. This is why we used different recruiting channels (university hospitals, general hospitals, practices, clinics, patient conferences, patient advocacy groups). Patients who take part in surveys and studies may be more committed, which might be a selection bias. It should be kept in mind that all results are patient-reported. Because the cohort consists of LTS, the treatment phase occurred many years ago, so there might be a recall bias.

Despite their descriptive nature and cross-sectional design, the results should be viewed as observational rather than definitive. Due to the absence of standardized long-term questionnaires, non-standardized questionnaires were used; to improve comparability, many questions were included from previous NOGGO Expression studies. As data were only gathered at a single time point, no dynamic evolution could be observed.

Ideally, a prospective trial would start from the initial diagnosis and follow patients for years also including a control group. While this would be interesting, it would also place a significant burden on patients and researchers, not to mention the high eventual dropout rates and costs.

### 4.2. Implication for Practice and Future Research

Our research showed a wide range of long-term side effects in gynecological cancer survivors, as well as an absence of evidence-based and standardized long-term follow-up care. Due to the wide range of issues that need to be considered in long-term follow-up care, this should not be left solely in the hands of outpatient gynecologists/primary care doctors. Instead, it should rather be managed by an interdisciplinary/multiprofessional team from various disciplines in cooperation with outpatient structures according to local infrastructure. Furthermore, the establishment of survivorship clinics for all types of gynecological cancer has the potential to enhance patient care by providing patients with a better understanding of their needs and offering them evidence-based information. However, large-scale prospective studies are needed to determine whether standardized long-term follow-up care actually improves the quality of life of LTS, such as the study “Survivorship Clinic” for long-term survivors with gynecological cancer (01NVF19005).

## 5. Conclusions

This study showed that long-term survivors (LTS) of gynecological tumors experience various symptoms and may undergo follow-up procedures that are not supported by current guidelines. These procedures may increase fears, invade privacy, and incur costs. It outlines the potential for implementing multidisciplinary and standardized long-term follow-up care. Just as surgical and medical treatment is more and more personalized, follow-up care should also be personalized—taking into account not only tumor type, but also age, comorbidities, frailty, etc. Furthermore, it is necessary to educate LTS about possible long-term side effects and lifestyle factors that have a positive influence on the overall outcome and quality of life, as well as about secondary cancers and how to prevent or detect them early.

## Figures and Tables

**Table 1:** The patients’ characteristics.

Patient Characteristics		n (%)
Age at initial diagnosis (n = 606)	<50 years	211 (34.8%)
	50–64 years	269 (44.4%)
	65–74 years	94 (15.5%)
	>75 years	32 (5.3%)
Overall survival (n = 495)	Median overall survival	7 years
		range: 5–38 years
	5–10 years survival	375 (75.8%)
	>10 years survival	120 (24.2%)
Tumor entity	Cervical cancer	277 (46.6%)
	Endometrial cancer	196 (32.9%)
	Ovarian, tubal, or peritoneal cancer	26 (4.4%)
	Other types of cancer	96 (16.1%)
FIGO stage (n = 508)	FIGO I	120 (23.6%)
	FIGO II	42 (8.3%)
	FIGO III	41 (8.1%)
	FIGO IV	11 (2.2%)
	Unknown to the patient	294 (57.9%)
Primary surgery (n = 627)	yes	589 (93.9%)
Chemotherapy (n = 625)	yes	238 (38.1%)
Radiotherapy (n = 622)	yes	245 (39.4%)
Maintenance therapy (n = 592)	yes	54 (9.1%)
Recurrent disease (n = 613)	yes	130 (21.2%)

**Table 2:** Long-term follow-up care percentages based on cases.

Follow-Up Procedure	All Cancer Types	Cervical Cancer	Endometrial Cancer	Ovarian/Tubal/Peritoneal Cancer	Other Cancer Patients
Not receiving any follow-up care	13.6%	16.9%	12.1%	7.7%	5.7%
Medical consultation	43.8%	38.6%	50.5%	30.8%	52.9%
Physical examination	49.7%	46.8%	54.7%	42.3%	49.4%
Serum CA-125	36.7%	30.7%	28.9%	80.8%	49.4%
Vaginal ultrasound	62.7%	62.2%	68.4%	50.0%	57.5%
Abdominal ultrasound	29.4%	28.5%	30.0%	38.5%	25.3%
PAP smear	40.6%	37.8%	50.5%	26.9%	41.4%
Clinical breast examination	52.8%	48.7%	58.9%	46.2%	48.3%
Breast ultrasound and/or mammography	40.4%	36.3%	45.3%	46.2%	34.5%
Imaging (e.g., MRI, CT, PET, chest X-ray, bone scintigraphy)	24.4%	23.6%	16.8%	53.8%	28.7%

## Data Availability

The datasets presented in this article are not readily available because the data are part of an ongoing study.

## Abbreviations

The following abbreviations are used in this manuscript:

LTS	Long-term survivors
